# Human APOE4 Protects High-Fat and High-Sucrose Diet Fed Targeted Replacement Mice against Fatty Liver Disease Compared to APOE3

**DOI:** 10.14336/AD.2023.0530

**Published:** 2024-02-01

**Authors:** Patricia Huebbe, Stephanie Bilke, Johanna Rueter, Anke Schloesser, Graeme Campbel, Claus-C. Glüer, Ralph Lucius, Christoph Röcken, Andreas Tholey, Gerald Rimbach

**Affiliations:** ^1^Institute of Human Nutrition and Food Science, Kiel University, D-24118 Kiel, Germany.; ^2^Institute of Experimental Medicine, Proteomics & Bioanalytics, Kiel University, D-24105 Kiel, Germany.; ^3^Section Biomedical Imaging, Department of Radiology and Neuroradiology, Kiel University, D-24118 Kiel, Germany.; ^4^Anatomical Institute, Kiel University, D-24118 Kiel, Germany.; ^5^Department of Pathology, Kiel University and University Hospital Schleswig-Holstein, Campus Kiel, D-24105 Kiel, Germany.

**Keywords:** hepatic proteome profiling, human apolipoprotein E, nonectopic lipid deposition, hepatic steatosis, steatohepatitis, NASH, hepatic pathogen recognition

## Abstract

Recent genome- and exome-wide association studies suggest that the human *APOE ε4* allele protects against non-alcoholic fatty liver disease (NAFLD), while *ε3* promotes hepatic steatosis and steatohepatitis. The present study aimed at examining the *APOE* genotype-dependent development of fatty liver disease and its underlying mechanisms in a targeted replacement mouse model. Male mice expressing the human APOE3 or APOE4 protein isoforms on a C57BL/6J background and unmodified C57BL/6J mice were chronically fed a high-fat and high-sucrose diet to induce obesity. After 7 months, body weight gain was more pronounced in human APOE than endogenous APOE expressing mice with elevated plasma biomarkers suggesting aggravated metabolic dysfunction. APOE3 mice exhibited the highest liver weights and, compared to APOE4, massive hepatic steatosis. An untargeted quantitative proteome analysis of the liver identified a high number of proteins differentially abundant in APOE3 versus APOE4 mice. The majority of the higher abundant proteins in APOE3 mice could be grouped to inflammation and damage-associated response, and lipid storage, amongst others. Results of the targeted qRT-PCR and Western blot analyses contribute to the overall finding that APOE3 as opposed to APOE4 promotes hepatic steatosis, inflammatory- and damage-associated response signaling and fibrosis in the liver of obese mice. Our experimental data substantiate the observation of an increased NAFLD-risk associated with the human *APOEε3* allele, while *APOEε4* appears protective. The underlying mechanisms of the protection possibly involve a higher capacity of nonectopic lipid deposition in subcutaneous adipose tissue and lower hepatic pathogen recognition in the APOE4 mice.

## INTRODUCTION

As a constituent of circulating lipoproteins, the apolipoprotein E (APOE) is known for its role to mediate lipoprotein binding to plasma membrane receptors enabling the exchange of lipids such as cholesterol between the periphery and the liver. Hence, the main production site of APOE is the liver, although other tissues and cells express significant amounts including activated macrophages and adipocytes. Beyond its classical role, APOE has been associated with the regulation of inflammatory processes [1, 2] and wound healing [3, 4], and with the outcome from acute infectious [5-8] and chronic inflammatory diseases [9-12], amongst others.

Two single nucleotide polymorphisms (SNP) in exon 4 of the human *APOE* gene distinguish between the three major allele variants *epsilon 4*, *epsilon 3* and *epsilon 2*. The rs429358(T) allele (*APOE epsilon 3*) is more common than the ancestral rs429358(C) allele (*APOE epsilon 4*). This SNP affects the amino acid residue 112 in the mature protein with an arginine-to-cysteine exchange from APOE4 to APOE3. The *APOE epsilon 4* allele has been associated with age-dependent morbidity and mortality and its frequency decreases significantly beyond the age of 80 [13-16]. An increased risk of cardiovascular disease (CVD) and Alzheimer’s disease (AD) with apparent gene-dosage effect has been accounted for the higher mortality of elderly *ε4* carriers [13-19]. However, this age-dependent disease risk seems to vary on different genetic backgrounds or facing different environmental conditions, such as high infection rates, leading also to comparably less depleted allele frequencies or even an accumulation of *APOE ε4* with increasing age in certain populations [20-22]. In addition to the adverse effects observed in the elderly, the *ε4* allele is suggested to exert beneficial effects in earlier life stages improving the outcome from recurring diarrhea, infant health status and cognitive development [23-28]. Under certain environmental conditions such as vitamin D insufficiency due to low endogenous synthesis, *APOE ε4* appears superior to the other alleles by increasing vitamin D status [29]. Furthermore, innate immune sensing, viral infection and hepatic stress response are modulated by the *APOE* variation with not just the *ε4* allele being disadvantageous [24, 30, 31, 32].

The human non-alcoholic fatty liver disease (NAFLD) is closely linked to obesity with emphasis on visceral adiposity, insulin resistance and the manifestation of the metabolic syndrome [33]. NAFLD includes liver steatosis and non-alcoholic steatohepatitis (NASH), with hepatocellular injury, lobular inflammation, fibrosis and cirrhosis as well as hepatocellular carcinoma as impending terminal complication [34]. While early observational studies investigating the relation of the *APOE* polymorphism and the risk of fatty liver disease, or NAFLD in particular, hardly find any significance [35-37], Sazci et al. reported an apparent accumulation of *APOE epsilon 3* homozygotes in NASH patients in 2008 [38]. This finding was then corroborated in case-control studies [39, 40] identifying *APOE epsilon 4* as the protective allele. Since then, several genome- and exome-wide association analyses have been confirming an association of the APOE4/APOE3-distinguishing SNP (rs429358) with liver function, liver fat content and fatty liver disease on different ethnic backgrounds [41-45]. The underlying mechanisms of this disease risk association, however, remain to be elucidated. *APOE* genotype-dependent differences in very low-density lipoprotein (VLDL) secretion, hepatic clearance of circulating lipids and immune cell activation may at least contribute to the increased risk of steatosis and steatohepatitis in non-*APOE epsilon 4* carriers.

Targeted replacement mice that express physiological levels of human *APOE* isoforms under the transcriptional control of the endogenous promoter are a suitable model to study gene-diet interactions at a molecular level [29, 46-50]. For example, comparable to the human age-dependent cognitive decline and Alzheimer’s disease pathology, APOE4 expressing mice display lower performances in spatial learning and memory tests [51, 52] with cellular and molecular alterations related to neurodegeneration [49, 53, 54]. Therefore, APOE targeted replacement mice provide a good opportunity to study the *APOE epsilon 3*-NASH-risk association at a physiological and molecular level. The present study aimed at the examination of the differential susceptibility to fatty liver disease development in the presence of human APOE3 as compared to APOE4 and its underlying metabolic and pathophysiological pathways.

## MATERIAL AND METHODS

### Mice and diet

The study was performed according to the German regulations of animal welfare and approved by the local animal ethical committee. The mouse experiment has been described also elsewhere [55-57]. In brief, twelve male APOE3 and APOE4 targeted replacement (six per *APOE* genotype group) and six unmodified C57Bl/ 6JBOM mice were purchased from Taconic Europe A/S (Ry, Denmark). The APOE targeted replacement has been developed on a C57Bl/6J background in the laboratory of Nobuya Maeda [58, 59] and described in more detail before [29, 46]. After the delivery at the age of 6-7 weeks, the mice were allowed to acclimate for two weeks before the study initiation. During this time, the mice were already fed with the experimental high-caloric diet. The semisynthetic diet was obtained from Ssniff (TD 88137 modified, Soest, Germany) and has been described before [46, 55]. The diet contained particularly high amounts of fat (21.2 %, from milk, with 0.2 % cholesterol) and sucrose (32.8 %, and 14.5 % starch from corn), which induces obesity, type 2 diabetes and metabolic syndrome in mice [60], and will be referred to as the high-fat and high-sucrose diet (HFSD) in the following. Daily, all mice were provided with fresh diet and drinking water. To minimize social stress due to rivalry, they were held individually in macrolon cages with environmental enrichment under a 12 h light-dark cycle and controlled environmental conditions (22-24 °C, 55-60 % humidity). Body weight was assessed weekly. One APOE4 mouse dropped out due to rapid significant weight loss and was euthanized four weeks before the completion of the study complying with the regulations of animal welfare. At the age of 9-10 months, all other mice were killed by cervical dislocation after individual admission of carbon dioxide and 4 hours of fasting. Blood was collected in heparinized tubes and the plasma was separated and stored in aliquots at -80 °C until use. The liver and spleen were removed, weighed, sectioned, snap frozen in liquid nitrogen and stored at -80 °C. Visceral adipose tissue was taken from the area surrounding the epididymis (referred to as epididymal white adipose tissue), snap frozen in liquid nitrogen and stored at -80 °C. For mRNA analysis of the liver, respective sections were put in RNAlater (Qiagen, Hilden, Germany) and stored at -20 °C until RNA isolation. For the histological evaluation of the liver, respective tissue sections were put in 4% para-formaldehyde until further processing.

### Analysis of plasma biomarkers

Plasma concentration of obesity-related metabolic biomarkers was measured with the following commercially available kits adhering to the manufacturer’s protocols: colorimetric assays from MedTest Dx (Canton, MI, USA) and Cayman (Ann Arbor, MI, USA) for triglycerides and glucose, ELISA kits from Merck Millipore (Darmstadt, Germany), R&D Systems (Abingdon, UK) and Abcam (Cambridge, UK) for insulin, leptin, and adiponectin, respectively. The HOMA-index was calculated as insulin [µU/ml] * glucose [mmol/l] / 22.5. The results for the unmodified C57BL/6J mice are according to [55]. Concentration of human APOE was determined with an ELISA kit from Abcam (ab108813, Berlin, Germany) according to the manufacturer’s instructions.

### Determination of subcutaneous and visceral adipose tissue (SAT and VAT) by micro-computed tomography (micro-CT)

SAT and VAT volumes were assessed in three mice per group at the age of 6 months using micro-CT, as described previously [55]. In brief, after the mice were anesthetized with Ketamine/Xylazine, the abdominal region between the first and the fifth lumbar vertebra was scanned using a conebeam *in vivo* micro-computed tomography system (vivaCT 40 Scanner, Scanco Inc., Bruettisellen, Suisse). The volume of SAT and VAT was calculated with an adapted algorithm [61] utilizing Canny Edge Detection and mathematical morphological operations (http://bme.sunysb.edu/labs/sjudex/miscellaneous.html). The results for SAT and VAT were related to the individual body weight of the mice at the time point of micro-CT analysis and given as % of total body weight. Data for the C57BL/6J mice are modified according to [55].

### Determination of energy expenditure by indirect calorimetry

Indirect calorimetry was performed with the TSE PhenoMaster (TSE Systems GmbH, Bad Homburg, Germany) measuring the volumes of O_2_ consumption (VO_2_) and CO_2_ production (VCO_2_) [55]. The indirect calorimetry was carried out at five different time points during the study period, adopting three mice per group. In order to reduce the stress level, those three out of six animals per group were selected for the indirect calorimetry that had not been assigned to the micro-CT analysis. Energy expenditure was determined using the following formula: (3.941*VO_2_ + 1.106*VCO_2_) *4.1868/1000 and expressed as kJ/(h*kg^0.75^). The results for the unmodified C57BL/6J mice are according to [55].

### Histopathological analysis

Liver sections of six APOE3 and APOE4 (three per group) were gelatin-embedded, cut into 5 µm thick slices and stained with hematoxylin and eosin (H&E) or Sirius Red. Liver tissue was visualized with a Zeiss Axio Observer D1 Inverted Fluorescence Microscope (Carl Zeiss Microscopy, Oberkochen, Germany) and representative images were selected. Histopathological scoring was conducted by a professional liver pathologist with profound experience in human NASH following the steatohepatitis scoring system proposed by Brunt et al. [62]. Briefly, H&E-stained liver tissues were reviewed for the grade of steatosis, lobular inflammation and ballooning. Results were then summarized in the NASH activity score (NAS) with 0-2 for definitively no steatohepatitis and 3-4 for possible steatohepatitis. A NAS of 5 or more would be applicable for definite steatohepatitis. The state of perisinusoidal and portal fibrosis was reviewed in Sirius Red stained sections with 0 standing for no fibrosis and 1a for perisinusoidal fibrosis, which is otherwise not visible in the H&E-stained tissue.

### Preparation of liver protein lysates from APOE3 and APOE4 mice

Total protein lysates were prepared in NP40 lysis buffer (150 mM NaCl, 50 mM Tris, 5 mM EDTA, 1 % NP40 substitute) containing a protease inhibitor cocktail, phenylmethylsulfonyl fluoride (both from Sigma-Aldrich®/Merck, Steinheim, Germany) and a phosphatase inhibitor (Roche, Mannheim, Germany). The liver tissue was homogenized with the TissueLyser II for 4 min at 25 Hz. Tissue lysates were incubated on ice for 30 min and centrifuged for 20 min, 4 °C at 12,000 x g. Supernatants (total protein) were stored in aliquots at -80°C until proteome analysis or Western blotting experiments. Nuclear and cytosolic extracts were prepared from fresh tissue as described elsewhere [63].

The protein concentration of liver lysates was determined photometrically by the bicinchoninic acid assay (BCA, Pierce Thermo Fisher Scientific, USA) on Tecan infinite 200 plate reader (Tecan, Grödig, Austria).

### Protein digestion and TMT-labelling (proteome analysis) of liver lysates

Liver lysates from three mice per group containing 100 µg protein were purified by chloroform-methanol-water precipitation. After resuspension of the precipitates in 20 µl of 0.25 % (w/v) RapiGest (Waters, Milford, MA, USA) in 100 mM triethylammonium bicarbonate (TEAB) buffer, sample volumes were adjusted to 100 µl with 100 mM TEAB. Reduction of disulfide bridges was performed using 5 µl tris (2-carboxyethyl) phosphine hydrochloride at 55 °C for 1 h and alkylation with 5 µl of 375 mM IAA at room temperature in the dark for 30 minutes. Sequencing grade modified trypsin (Promega, Madison, WI, USA) was added at a 1:50 protease to protein ratio and incubated overnight at 37 °C. Peptides were labelled with TMT-6plex according to the vendors protocol (Thermo, Bremen, Germany), with a TMT to peptide ratio of 1:4 [64]. The six labelled samples within an experiment were pooled equally in a new lobind reaction tube (Eppendorf, Hamburg, Germany), acidified by addition of 10 % trifluoroacetic acid and desalted using C18 Sep-Pak Vac (1cc/50 mg, Waters). Using a vacuum centrifuge, the combined samples were dried and resuspend in 60 µl of eluent A (see below) for high-performance liquid chromatography (HPLC) separation at pH 10.

### Peptide fractionation, LC-MS analysis and database search (proteome analysis)

A two-dimensional, semi-orthogonal scheme, consisting of a reversed-phase HPLC at pH 10 and an ion-pair reversed-phase HPLC at pH 2 was used for peptide separation [65]. An Ultimate 3000 HPLC system (Dionex, Dreieich, Germany) with a *Gemini*-C18 column (250 mm x 3 mm, 3 µm; Phenomenex, Aschaffenburg, Germany) was used for separation in the first dimension. Eluent A: 72 mM triethylamine in milliQ water, pH 10 with acetic acid; eluent B: 72 mM triethylamine in acetonitrile, 0.35% (v/v) acetic acid. Approximately 250 µg of labelled peptides were washed at a flow rate of 200 µl/min by a gradient from 2 to 5% B in 5 min and then fractionated over a linear gradient to 55% B in 50 min. After a linear increase to 95% B in 5 min and a 5 min washing step, the column was equilibrated at 5% B for an additional 15 min. From minute two, fractions were collected every minute up to a total of 56 fractions. The elution profile was monitored with a UV detector at 280 nm. The fractions were dried in vacuum, resuspended in 20 µl in 3 % acetonitrile, 0.1% trifluoroacetic acid and pooled into seven fractions according to the following scheme: pool 1: fractions 1, 8, 15, 22 ,29, 36, 43, 50; pool 2: fractions 2 to 51; respectively.

For second dimension separation, an Ultimate 3000 nano-HPLC system with an Acclaim PepMap100 nano-column (75 µm x 15 cm, 3 µm, 100 Å; Dionex) coupled online to a Q Exactive HF Orbitrap mass spectrometer (Thermo) was used. Eluent A: 0.05% formic acid (FA); eluent B: 0.05 % FA, 80 % acetonitrile. A spray voltage of 2.1 kV and a capillary temperature of 250 °C were used. Per pooled fraction 4 µl were injected, separation was performed with a 210 min total gradient at a flow rate of 300 nl/min. A linear gradient from 4 to 40% B in 182 min, followed by a linear increase to 90 % B in 5 min, a 10 min wash step, and 13 min of equilibration time with 4% B was used. Additionally, separation was monitored at 214 nm. MS1 spectra (scan range 375-1400 *m/z*) were recorded at a resolution of 60k, with an automatic gain control (AGC) target of 3E6 and maximum inject time of 100 ms. The 10 most intense precursors were selected for fragmentation at MS2 level at a resolution of 15k, an AGC target of 1e5, a maximum injection time of 100 ms and with an isolation window of 1.2 *m/z*. The normalized collision energy was set to 33 and the dynamic exclusion to 20 s. Each fraction was analyzed in three technical replicates by LC-MS/MS.

Database search and peptide quantification were performed using Proteome Discoverer Software (v2.2.0.388) and searched against the mouse reference proteome (55,493 entries, downloaded on June 16, 2021) supplemented with the human sequence of APOE3 and APOE4 (downloaded on August 05, 2021) and common contaminants (cRAP, 116 entries, downloaded on May 04, 2021). SequestHT search algorithm was used with a fragment mass tolerance of ± 0.02 Da, precursor mass tolerance of ± 8 ppm and with up to two allowed missed cleavages. Oxidation was set as a variable, carbamidomethylation and TMT6plex (N-term and K) were set as static modifications. For quantification razor and unique peptides were used. Statistic and functional annotation were performed using Perseus Software (v.1.6.15.0). Two-sided Student’s t-test with an artificial variance (S0) of 0.3 was applied to the log_2_ transformed protein-ratios to test the biological conditions against each other. The permutation-based false discovery rate (FDR) approach with 250 randomizations (FDR 0.05) was used to correct the p-values. The quantified proteins were annotated with gene ontology and annotations were used for Fisher exact test and one-dimensional annotation enrichment test (FDR 0.05).

LC-MS data have been deposited to the ProteomeXchange Consortium [66] via PRIDE partner repository with the dataset identifier PXD033973.

### Western blotting with liver lysates

Liver protein lysates were loaded on TGX Stain-Free Precast gradient gels (Biorad, Munich, Germany) (40 µg protein per lane) and separated by SDS precast polyacrylamide gel electrophoresis, as described in detail in previous publications [55, 63]. Following the protein transfer onto a polyvinylidene difluoride membrane with the Trans Blot Turbo™ System (Biorad), the target proteins were identified using the following primary monoclonal antibodies (all purchased from Merck): anti-phospho-cytokeratin-8 (Ser73) (clone LJ4), anti-cytokeratin 8 (clone TROMA-1) and anti-HMGB1 (clone 3K6). Secondary horseradish peroxidase-conjugated antibodies were purchased from Biorad (Immun-star goat anti-mouse IgG (1705047) and anti-rabbit IgG (1705046)) and Merck (anti-rat IgG (A9037)). Target bands were visualized by chemiluminescent ECL Western blot substrates (Fisher Scientific, Schwerte, Germany) in a ChemiDoc XRS system (Biorad) and band intensity was analyzed using the Image Lab 4.1 software (Biorad). Target proteins were normalized by the total protein load per lane, measured as membrane fluorescence and as described previously [55]. The determination of hepatic APOE has been performed in a previously published study and the values are according to Rueter et al. [57].

**Table 1:** Primer sequences used for the qRT-PCR analyses in mouse liver and visceral adipose tissue.

Gene	Product	Forward 5’-3’	Reverse 5’-3’
***Acta2***	Actin, aortic smooth muscle	AACACGGCATCATCACCAAC	AGTGTCGGATGCTCTTCAGG
***Adgre1***	Adhesion G protein-coupled receptor E1 (F4/80)	CAGGGCAGGGATCTTGGTTA	ACACTGGGGCACTTTTGTTC
***Adipoq***	adiponectin	AGACCTGGCCACTTTCTCCT	ACGTCATCTTCGGCACT
***APOE***	human apolipoprotein E	ACCCAGGAACTGAGGGC	CTCCTTGGACAGCCGTG
***Pnpla2***	patatin-like phospholipase domain containing 2 (Atgl)	AGCATCCAGTTCAACCTTCG	TTGGTTCAGTAGGCCATTCC
***Ccl2***	chemokine (C-C motif) ligand 2	CATCCACGTGTTGGCTCA	GATCATCTTGCTGGTGAATGAGT
***Cd2***	T-cell surface antigen CD2	GACTAGGCTGGAGAAGGACC	TCAAAATCTGTCCCTTGCAAGA
***Cd3g***	CD3 antigen, gamma polypeptide	GCCTCTTCCTGGTGATCTCT	GTCAGTCAAGCCACAAGTCA
***Cd36***	CD36 molecule	CAAAACGACTGCAGGTCAAC	CCAATGGTCCCAGTCTCATT
***Cd40***	Tumor necrosis factor receptor superfamily member 5	GGCCACTGAGACCACTGATA	CGCATCCGGGACTTTAAACC
***Cd68***	macrosialin	CAGGACCTACATCAGAGCCC	TTCTGCGCCATGAATGTCCA
***Cd163***	Scavenger receptor cysteine-rich type 1 protein M130	ATGCTTCCATCCAGTGCCT	GGCTCCACAAACCAAGAGTG
***Col1a1***	Type I collagen	TTCACCTACAGCACCCTTGT	AGTCCGAATTCCTGGTCTGG
***Des***	Desmin	ACCAGATCCAGTCCTACACC	AGCTCCCTCATCTGCCTCA
***Fasn***	Fatty acid synthase	AAGGCTGGGCTCTATGGATT	TGAGGCTGGGTTGATACCTC
***Gt2b***	General transcription factor IIB	TGGAGATTTGTCCACCATGA	AGGTTGATTCTGTCCGCCAT
***Icam1***	Intercellular adhesion molecule 1	AGGGCTGGCATTGTTCTCTA	CCAGGGAGCAAAACAACTTC
***Ldlr***	low density lipoprotein receptor	CCTCAAGATTGGCTCTGAGTG	GCCTGGCACTCACACTTGTA
***Lep***	Leptin	TCATTGGCTATCTGCAGCAC	TGACACCAAAACCCTCATCA
***Mmp13***	Collagenase 3	GGCCACCTTCTTCTTGTTGA	GGTGGGATCATCAAGTTT
***Mpo***	Myeloperoxidase	CTTTGACAGCCTGCACGATG	CAAAGAGGGTGTGCATGGAG
***Pnpla2***	patatin-like phospholipase domain containing 2	AGCATCCAGTTCAACCTTCG	TTGGTTCAGTAGGCCATTCC
***P2rx7***	P2X purinoceptor 7	GAAAGAAGCTCCCCGACCT	TGGCAAGATGTTTCTCGTGG
***Rn18S***	18S ribosomal RNA	GGTAACCCGTTGAACCCCAT	CAACGCAAGCTTATGACCCG
***Tgfb1***	Transforming growth factor beta-1	TCACTGGAGTTGTACGGCAG	ATCCCGTTGATTTCCACGTG
***Timp1***	Metalloproteinase inhibitor 1	TCCCCAGAAATCAACGAGAC	AAGAAGCTGCAGGCACTGAT

### RNA isolation and quantitative reverse transcriptase polymerase chain reaction (qRT-PCR)

Total RNA from the liver was isolated using the miRNA NucleoSpin® Kit (Macherey & Nagel, Düren, Germany) and quantified as described previously [32, 55]. Total RNA from epididymal white adipose tissue (VAT) was isolated by optimized guanidine isothiocyanate/phenol-chloroform extraction using peqGOLD TriFast^TM^ (VWR International, Leuven, Belgium). The primers for the qRT-PCR were designed using Primer3 Input software version 4.1.0 (https://primer3.ut.ee/) and purchased from Eurofins MWG (Ebersberg, Gemany) (Table 1). The qRT-PCR was performed using the SensiMix™ SYBR No-ROX one step kit (Bioline, Luckenwalde, Germany) on a Rotorgene 6000 cycler (Corbett Life Science, Sydney, Australia). Target gene mRNA expression was calculated using an external standard curve and related to the housekeeper *Rn18S* (for liver) or the mean of *Rn18S* and *Gt2b* (for VAT).

### Statistical analysis

Statistical analysis of all data except for the results of the proteome analysis was conducted with GraphPad Prism 9.5.1 (GraphPad Software, USA). Data were analyzed for normality of distribution with the Shapiro-Wilk test and the homogeneity of the variances was tested with the Brown-Forsythe test. An ordinary one-way ANOVA was applied for the comparison of APOE3, APOE4 and C57BL/6J mice with a Fisher’s LSD test for multiple comparisons. In the absence of normally distributed data, a Kruskal Wallis ANOVA was performed. For the comparison of two groups only (APOE3 vs. APOE4), an unpaired Student’s *t*-test or a nonparametric Mann-Whitney U test (for non-normally distributed data) was performed. Statistical outliers were identified applying the Grubb’s test with an α-value of 0.001. Individual results, means and SEM are shown. Significance was accepted at p<0.05 and indicated with an asterisk.

**Figure 1.**
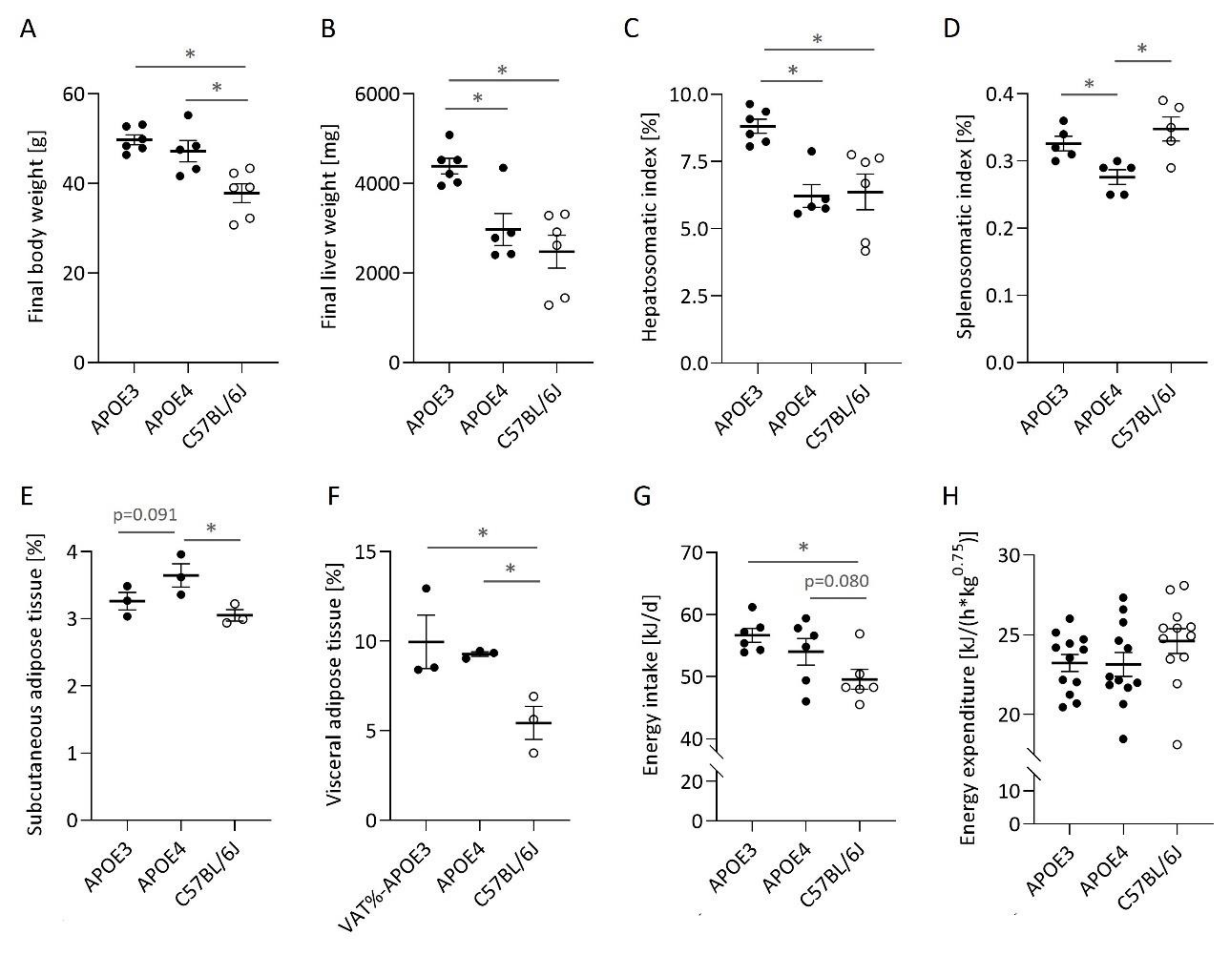
**Human APOE expressing mice develop more pronounced obesity on a high-fat and high-sucrose diet compared to C57BL/6J mice, with apparent liver enlargement evident in APOE3 mice**. The final body weight (A) and liver (B) weights of APOE3 and APOE4 TR mice compared to C57BL/6J mice with similar genetic background were assessed after 7 months of HFSD feeding. Relative liver and spleen weights expressed as percent final body weight are given as hepatosomatic (C) and splenosomatic index (D). After 6 months, the body composition of three representative mice per group was assessed using micro-computed tomography. The subcutaneous (E) and visceral (F) adipose tissue volumes are given as percent of the actual body weight. Individual daily food intake was monitored over the whole feeding period and expressed as mean energy intake (G). Total energy expenditure (EE) was calculated using the individual oxygen consumption and carbon dioxide production per hour measured over a period of 24 hours using indirect calorimetry. Three mice per group were subjected to indirect calorimetry at four consecutive time points throughout the feeding period. The mean total EE per mouse and time point are depicted (H). Individual data, means and the SEM are shown. For A-D, all animals per group were included in the analysis (APOE3 and C57BL/6, n=6; APOE4, n=5) and shown except for one statistical outlier in D (C57BL/6J). An asterisk indicates a statistically significant difference (p<0.05) between two groups (indicated by the bar) detected by an ordinary one-way ANOVA or a non-parametric Kruskal Wallis test (C, F) following a Fisher’s LSD test for multiple comparisons. In A, E-F and H, values for C57BL/6J mice were according to Schloesser et al. [55]. Final liver weight of APOE3 and APOE4 mice (B) is according to Dose et al. [32].

## RESULTS

### Diet-induced obesity (DIO) was more pronounced in human APOE expressing mice compared to C57B/6J counterparts, but the liver enlargement was highest in APOE3 mice

All animals developed an obese phenotype after long-term feeding with the HFSD, which was significantly more pronounced in human APOE expressing mice (Fig. 1A). The final body weight was, however, not significantly different between APOE3 and APOE4 mice, given the very high inter-individual variance in the latter group (Fig. 1A). Liver weights were disproportionally increased in APOE3 mice (Fig. 1B), which is reflected by the still significantly higher hepatosomatic index, relating the liver to the body weight (Fig. 1C). The spleens were also enlarged in APOE3 mice (181 ± 21 mg) compared to the other groups (131 ± 10 mg (APOE4) and 137 ± 11 mg (C57BL/6J)). However, related to the body weight, the splenosomatic index (Fig. 1D) was significantly lowest in APOE4 compared to APOE3 and C57BL/6J mice.

Similar to the final body weight, the relative total abdominal fat mass of human APOE expressing mice was higher compared to C57BL/6J mice (13.2 ± 1.60 % and 12.9 ± 0.25 % compared to 8.5 ± 0.98 %, p=0.074 and p=0.025) with no significant difference between APOE3 and APOE4. While visceral adipose tissue was not significantly different between APOE3 and APOE4 mice (Fig. 1F), subcutaneous adipose tissue percentage tended (p=0.091) to be higher in APOE4 (Fig. 1E).

Human APOE3 and APOE4 mice were equally hyperphagic and exhibited a higher calorie intake than C57BL/6J mice (Fig. 1G), while total energy expenditure was not significantly different between the groups (Fig. 1H).

**Table 2:** mRNA levels of genes encoding proteins involved in the regulation of metabolic processes and inflammation in the visceral adipose tissue of mice expressing either human APOE (APOE3 and APOE4) or endogenous mouse APOE (C57BL/6J).

Gene	Description of biological function	mRNA level			Individual p-value		
		APOE3	APOE4	C57BL/6J	APOE3 vs. APOE4	APOE3 vs. C57BL/6J	APOE4 vs. C57BL/6J
***Adipoq***	Adiponectin, promotes glucose uptake, positive metabolic regulation, attenuation of cellular immunity	1.59 ± 0.25	2.32 ± 0.34	3.46 ± 0.29	*0.099*	*0.0003*	*0.017*
***Ccl2***	Chemoattractant cytokine for monocytes	3.09 ± 0.37	2.80 ± 0.72	1.38 ± 0.23	*0.671*	*0.016*	*0.046*
***Tnfa***	Proinflammatory cytokine	7.45 ± 1.10	4.32 ± 1.04	4.50 ± 1.25	*0.070*	*0.085*	*0.915*
***Adgre1***	F4/80, marker of tissue macrophages	3.64 ± 0.50	2.61 ± 0.48	1.85 ± 0.33	*0.128*	*0.011*	*0.252*
***Cd163***	Surface receptor expressed on M2-type macrophages	1.21 ± 0.03	1.30 ± 0.14	2.74 ± 0.61	*0.880*	*0.019*	*0.026*
***Cd36***	Lipoprotein binding, lipid uptake, lipolysis, preadipocyte recruitment	1.54 ± 0.19	1.92 ± 0.25	2.27 ± 0.20	*0.240*	*0.027*	*0.280*
***Pnpla2***	Adipose tissue triglyceride lipase, cellular triglyceride catabolism	2.37 ± 0.28	2.18 ± 0.48	5.20 ± 1.13	*0.860*	*0.017*	*0.015*
***Fasn***	Fatty acid synthase, triglyceride anabolism	1.52 ± 0.12	1.92 ± 0.43	2.07 ± 0.36	*0.402*	*0.232*	*0.749*
***Ldlr***	LDL receptor, apolipoprotein E-dependent lipoprotein binding, lipid uptake	0.87 ± 0.09	1.11 ± 0.14	0.81 ± 0.08	*0.114*	*0.695*	*0.058*
***Lep***	Leptin, positive regulation of insulin and cytokine secretion, promotion of cellular immunity	2.13 ± 0.16	2.10 ± 0.33	1.20 ± 0.27	*0.917*	*0.018*	*0.028*

mRNA levels of target genes were related to the mean expression of two housekeeping genes (*Gt2b, Rn18s*). Data are means ± SEM (n=5-6). Ordinary one-way ANOVAs were performed with an uncorrected Fisher’s LSD test comparing each group with every other group and individual p-values are given

### No APOE genotype-dependent effect on plasma biomarkers of metabolic function after long-term HFSD feeding

**Figure 2.**
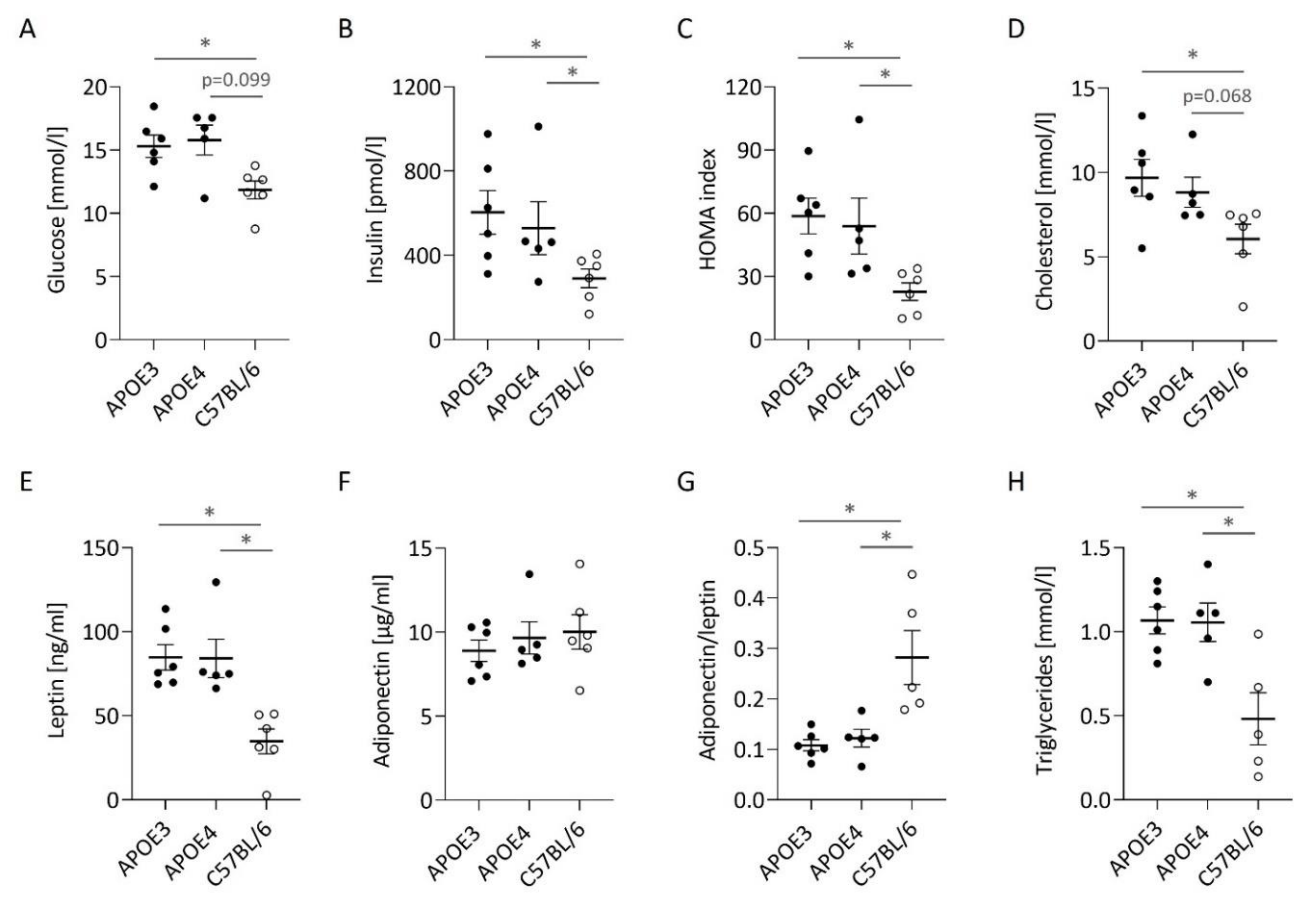
**Obesity in human APOE expressing mice is associated with the aggravation of circulating metabolic biomarkers compared to unmodified C57BL/6J mice, but without any difference between APOE3 and APOE4**. Concentration of glucose (A), insulin (B), cholesterol (D), leptin (E), adiponectin (F) and triglycerides (H) was measured in the plasma of four hour-fasted animals chronically fed a high-fat and high-sucrose diet. The HOMA index (C) was calculated as insulin [µU/ml]*glucose [mmol/l]/22.5 and adiponectin/leptin (G) as the ratio of adiponectin [µg/ml] to leptin [ng/ml]. Individual data, means and the SEM are shown. All animals per group were analyzed (APOE3 and C57BL/6, n=6; APOE4, n=5) and shown except for one statistical outlier in G and H (C57BL/6J). An asterisk indicates a statistically significant difference (p<0.05) between two groups (indicated by the bar) detected by an ordinary one-way ANOVA or a non-parametric Kruskal Wallis test (E, F) following a Fisher’s LSD test for multiple comparisons. The values for C57BL/6J mice were according to Schloesser et al. [55] with slight modifications for A-D and G-H (conversion into SI units).

With the exception of adiponectin, obesity-related biomarkers including glucose, insulin, triglycerides, cholesterol, and leptin were significantly aggravated in human APOE expressing mice compared to unmodified C57BL/6J mice (Fig. 2A-H). The calculated ratios, the HOMA-index and adiponectin-to-leptin, displayed a significant deterioration in metabolic function in targeted replacement mice (Fig. 2C and 2G). However, there were no differences observable between the two *APOE* genotype groups.

### Differences in mRNA levels in the visceral adipose tissue are significant between human APOE3 expressing mice and unmodified C57BL/6J mice

Most of the measured obesity-related genes were altered in human APOE expressing mice, whereby the difference was greater and thus significant only comparing APOE3 with C57BL/6J mice (Table 2). The most apparent effects on mRNA levels were observed for adiponectin (*Adipoq*), *Ccl2*, *TNFa* and the macrophage marker F4/80 (*Adgre1*) indicating an increased level of adipose tissue inflammation associated with human APOE3 (and less with human APOE4) expression as compared to unmodified C57BL/6J mice. The mRNA levels of markers of adipocyte proliferation and adipocyte-specific metabolism such as *Cd36* and *Pnpla2* were significantly lower in APOE3 than C57BL/6J mice. There were no significant differences between the two *APOE* genotype groups apart from in trend lower levels of *Adipoq* and *TNFa* mRNA in APOE4 mice.

### Evidence of fatty liver disease in APOE3 compared to APOE4 mice

**Figure 3.**
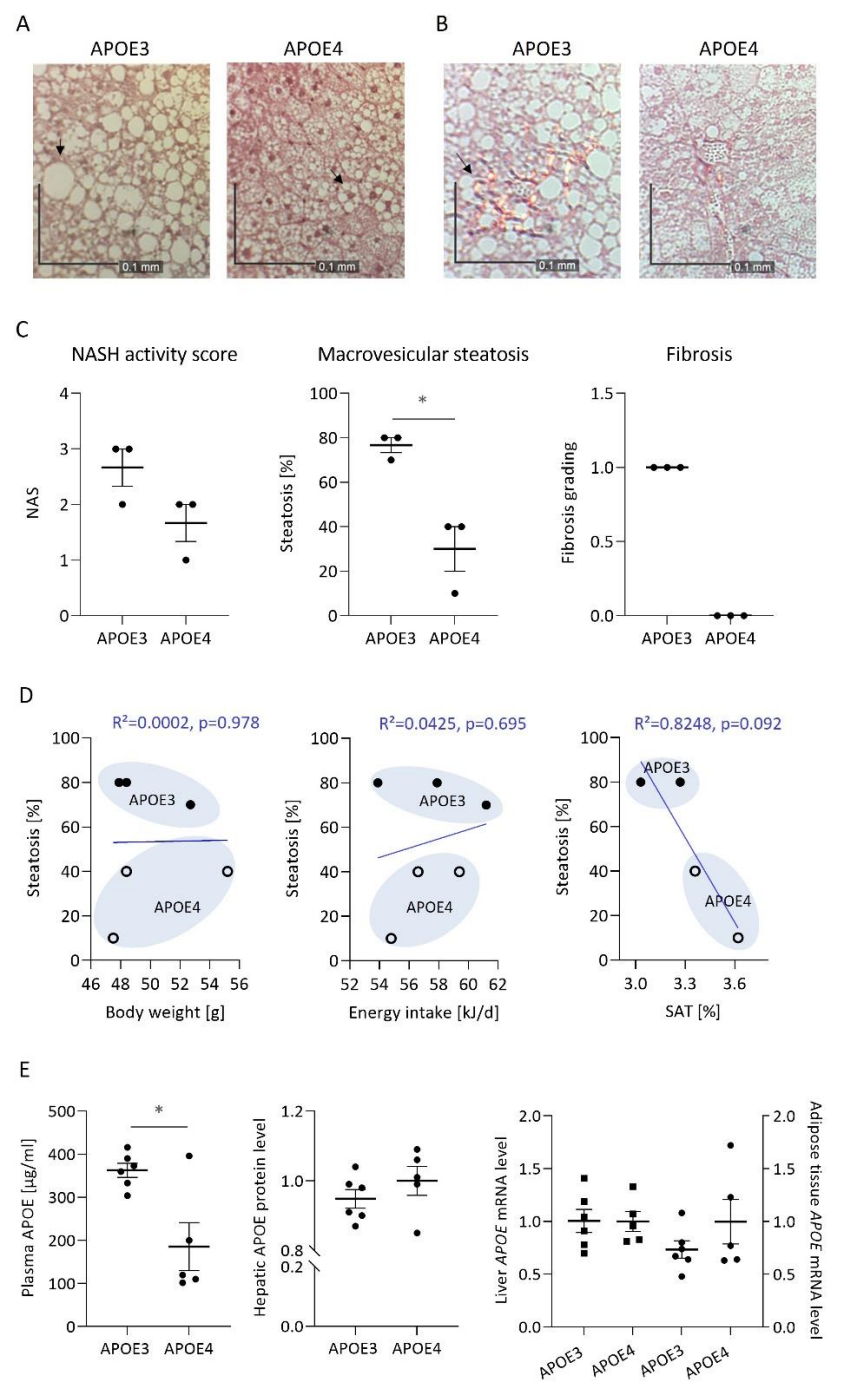
**Expression of the human APOE3, as compared to APOE4, leads to severe hepatic steatosis and initial perisinusoidal fibrosis, which is not a function of body weight or energy intake in obese targeted replacement mice**. Liver sections of three representative APOE3 and APOE4 mice were stained with hematoxylin and eosin (A) and Sirius Red (B) to visualize hepatic lipid deposition and collagen production. Representative images were obtained with 100x magnification. Black arrows indicate differentially sized lipid-containing hepatocytes (A) or collagen indicating fibrosis (B). The liver sections were evaluated by a professional liver pathologist, who calculated the NASH activity score (NAS) including the quantification of macrovesicular steatosis and fibrosis (C). Individual data, the mean of the group and the SEM are displayed (n=3). Simple linear regressions (D) were performed testing the percentage of hepatic steatosis as variable depending on either the body weight (plot on the left), the daily energy intake (plot in the middle) or the percentage of subcutaneous adipose tissue (SAT related to the body weight) (plot on the right). The plots show individual data pairs of APOE3 mice (closed circles) and APOE4 mice (open circles), as well as the trend line, R squared and p-values of the regression. (**E**), the circulating APOE concentration was determined in the plasma of 4 h fasted mice after termination of the feeding trial using an ELISA kit. Hepatic APOE protein levels were analyzed by Western blotting and the data shown are according to Rueter et al. [57]. Relative mRNA levels of human *APOE* were analyzed in the liver (squares) and visceral adipose tissue (circles) by qRT-PCR. Target gene expression was related to housekeeping gene expression (*Rn18S* in the liver and the mean of *Rn18S* and *Gt2b* in the VAT). Individual data, means and SEM were shown (APOE3, n=6; APOE4, n=5). An asterisk indicates a statistically significant difference between APOE3 and APOE4 determined by the Student’s t-test (p<0.05).

We observed massive hepatic lipid deposition in APOE3 mice visible in the H&E-stained liver sections, while hepatic steatosis was less pronounced in APOE4 mice (Fig. 3A). Corresponding hepatic macrovesicular steatosis scores were significantly higher in APOE3 than APOE4 mice (Fig. 3C). Initial perisinusoidal fibrosis was visible in Sirius Red stained APOE3 but not in APOE4 liver sections (Fig. 3B). Added together, APOE3 mice reached a higher NASH activity score compared to APOE4 (Fig. 3C). A simple linear regression was performed confirming the *APOE* genotype as explanatory variable of hepatic steatosis (R^2^=0.8305, p=0.012). A higher liver weight was accompanied by a higher degree of hepatic steatosis, but statistical significance was not reached (R^2^=0.4853, p=0.124). Furthermore, correlations with the body weight, energy intake and percentage of SAT were calculated (Fig. 3D), but neither of them was a significant predictor of hepatic steatosis. In contrast to the other variables, percentage of SAT and steatosis show an inverse relationship. Due to the fact that histological examination of the liver and assessment of the body composition have not been conducted in all animals per group, this regression model is based on only four data pairs, which hampers statistical significance of the mean difference. Plasma APOE levels were significantly higher in APOE3 than APOE4 mice despite similar hepatic protein levels and mRNA levels in the liver and VAT (Fig. 3E). There is a significant association of plasma APOE and final body weight (R^2^=0.4803, p=0.018). In contrast to the *APOE* genotype, the level of plasma APOE is not a significant explanatory variable of hepatic steatosis in our mice (R^2^=0.4141, p=0.168).

**Table 3:** Biological function of the 105 proteins with increased abundance in the liver of APOE3 compared to APOE4 mice.

Biological function	Protein description	Gene
**Lipid storage and metabolism**	Apolipoprotein A-IV	*Apoa4*
	Apolipoprotein E	*Apoe*
	Fatty acid-binding protein, adipocyte	*Fabp4*
	Fatty acid-binding protein, brain	*Fabp7*
	Perilipin-3	*Plin3*
	Perilipin-4	*Plin4*
	Chemerin	*Rarres2*
**Glycolysis**	Fructose-bisphosphate aldolase	*Aldoa*
	Alpha-enolase	*Eno1*
	Glucose-6-phosphate isomerase	*Gpi*
	Pyruvate kinase PKM	*Pkm*
**Pentose-phosphate shunt and nucleotide synthesis**	Adenine phosphoribosyltransferase	*Aprt*
	UMP-CMP kinase	*Cmpk1*
	6-phosphogluconate dehydrogenase, decarboxylating	*Pgd*
**Cytoskeleton remodeling, regulation of cell migration**	Rho GDP-dissociation inhibitor 1	*Arhgdia*
	Biglycan	*Bgn*
	Collagen alpha-1(XIV) chain	*Col14a1*
	Coactosin-like protein	*Cotl1*
	Cytoglobin	*Cygb*
	Dystroglycan	*Dag1*
	Ena/VASP-like protein (Fragment)	*Evl*
	Ras GTPase-activating-like protein IQGAP1	*Iqgap1*
	Keratin, type I cytoskeletal 18	*Krt18*
	Keratin, type II cytoskeletal 8	*Krt8*
	Lumican	*Lum*
	Myristoylated alanine-rich C-kinase substrate	*Marcks*
	MARCKS-related protein	*Marcksl1*
	Plastin-1	*Pls1*
	Prolargin	*Prelp*
	Transgelin-2	*Tagln2*
	Protein-glutamine gamma-glutamyltransferase 2	*Tgm2*
	Tubulin beta-6 chain	*Tubb6*
	Vimentin	*Vim*
	14-3-3 protein eta	*Ywhah*
**Xenobiotic metabolism, phase I and II**	Carbonyl reductase [NADPH] 1	*Cbr1*
	Carbonyl reductase [NADPH] 3	*Cbr3*
	Glutathione synthetase	*Gss*
	NAD(P)H dehydrogenase [quinone] 1	*Nqo1*
	UDP-glucuronosyltransferase 1A9	*Ugt1a9*
**Endopeptidases and inhibitors**	Cystatin-B	*Cstb*
	Cathepsin D	*Ctsd*
	Serine protease inhibitor A3K	*Serpina3k*
	Serine protease inhibitor A3M	*Serpina3m*
	Serine (or cysteine) peptidase inhibitor, clade B, member 6a	*Serpinb6a*
	Serine protease inhibitor H1	*Serpinh1*
**Immune response**	CD5 antigen-like	*Cd5l*
	Macrosialin	*Cd68*
	H-2 class II histocompatibility antigen gamma chain	*Cd74*
	CTP synthase 1	*Ctps1*
	Cytochrome b-245 heavy chain	*Cybb*
	High affinity immunoglobulin epsilon receptor subunit gamma	*Fcer1g*
	Hematopoietic lineage cell-specific protein	*Hcls1*
	Haptoglobin	*Hp*
	Interferon alpha-inducible protein 27-like protein 2B	*Ifi27l2b*
	Immunoglobulin heavy constant alpha (Fragment)	*Igha*
	Immunoglobulin heavy constant gamma 2B (Fragment)	*Ighg2b*
	Immunoglobulin heavy constant gamma 2C (Fragment)	*Ighg2c*
	Integrin beta-2	*Itgb2*
	Galectin-1	*Lgals1*
	Galectin-3	*Lgals3*
	Lysozyme C-2	*Lyz2*
	Interferon-activable protein 205-B	*Mnda*
	Bone marrow proteoglycan	*Prg2*
	Protein S100-A1	*S100a1*
	Protein S100-A10	*S100a10*
	Protein S100-A11	*S100a11*
	Zinc transporter ZIP4	*Slc39a4*
	WD repeat and FYVE domain-containing protein 1	*Wdfy1*
	Ig heavy chain V region 6.96	
	Ig heavy chain V region AC38 205.12	
**Tissue damage associated and ER stress response**	Annexin A2	*Anxa2*
	Annexin A3	*Anxa3*
	Annexin A5	*Anxa5*
	F-box only protein 6	*Fbxo6*
	Fructosamine-3-kinase	*Fn3k*
	Progranulin	*Grn*
	High mobility group AT-hook protein 1	*Hmga1*
	High mobility group nucleosome-binding domain-containing protein 2	*Hmgn2*
	Heat shock protein beta-1	*Hspb1*
	Mixed lineage kinase domain-like protein	*Mlkl*
	Signal transducing adapter molecule 1	*Stam*
	Thymosin beta-4	*Tmsb4x*
**Miscellaneous**	Lysosomal acid phosphatase	*Acp2*
	Ataxin-2-like protein	*Atxn2l*
	Brain acid soluble protein 1	*Basp1*
	BET1-like protein	*Bet1l*
	CKLF-like MARVEL transmembrane domain-containing protein 4	*Cmtm4*
	Histone H1.1	*H1-1*
	Histone H1.5	*H1-5*
	Histone H2A type 1-K	*H2ac15*
	BTB/POZ domain-containing protein KCTD12	*Kctd12*
	Lymphatic vessel endothelial hyaluronic acid receptor 1	*Lyve1*
	Mitofusin-1	*Mfn1*
	Major vault protein	*Mvp*
	Neudesin	*Nenf*
	Diphosphoinositol polyphosphate phosphohydrolase 2	*Nudt4*
	Retinol-binding protein 1	*Rbp1*
	RNA-binding protein with multiple splicing	*Rbpms*
	Non-specific serine/threonine protein kinase (Fragment)	*Rps6ka1*
	Cornifin-A	*Sprr1a*
	THUMP domain-containing protein 3	*Thumpd3*
	Transmembrane protein 176B	*Tmem176b*
	Thymidine phosphorylase	*Tymp*
	UDP-N-acetylhexosamine pyrophosphorylase-like protein 1	*Uap1l1*
	UPF0688 protein C1orf174 homolog	

### Large differences in hepatic protein abundance between APOE3 and APOE4 mice

**Figure 4.**
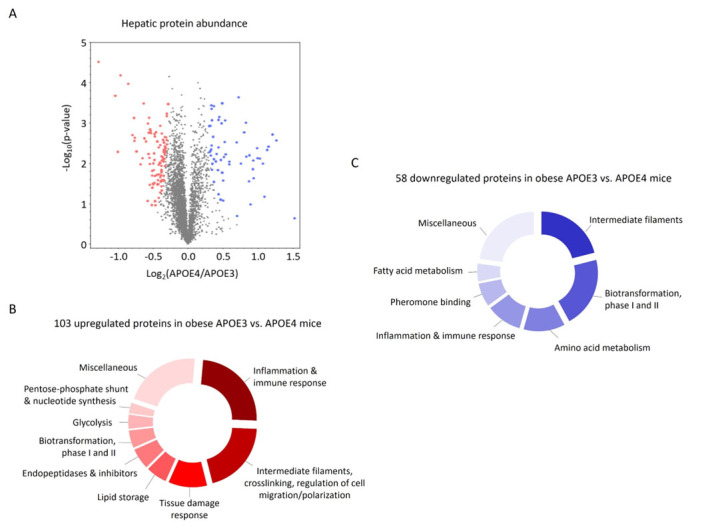
**Hepatic steatosis leads to large differences in protein abundance in obese human APOE3 compared to APOE4 expressing mice**. Protein abundance in the liver was determined in three mice per group by proteome analysis using LC-MS. The volcano plot depicts the identified proteins in APOE4 versus APOE3 mice, and the significant proteins are highlighted in red and blue (A). Significantly higher (B) and lower (C) abundant proteins in APOE3 mice were then grouped according to their biological function.

The liquid chromatography-mass spectrometry (LC-MS) analysis of hepatic protein abundance revealed large differences between APOE3 and APOE4 mice (Fig. 4A). We observed 105 proteins with higher and 59 proteins with lower abundance in APOE3 mice (Tables 3 and 4). APOE3 is, incidentally, among the higher abundant proteins in APOE3 expressing mice. However, in contrast to the identification of APOE3 and APOE4, a specific differentiation in the quantification is not possible due to the high similarity of the isoforms and the applied search settings of the database searches. Therefore, the level of hepatic APOE has been examined in an independent experiment using Western blotting (Fig. 3E).

The majority of the proteins with higher abundance in APOE3 mice can be grouped to i) immune cell function and inflammatory responses (25 proteins), ii) intermediate filaments, crosslinking and the regulation of cell migration or polarization (21 proteins), iii) tissue damage associated and ER stress response (11 proteins), and iv) lipid storage (7 proteins) (Fig. 4B). In contrast, proteins related to normal “healthy” liver function appear to be higher abundant in APOE4 mice. Those proteins can be grouped to i) xenobiotic metabolism and biotrans-formation (12 proteins), ii) amino acid metabolism (7 proteins) and iii) the production of proteins for pheromone binding (4 proteins), amongst others (Fig. 4C).

**Table 4:** Biological function of the 59 proteins with decreased abundance in the liver of APOE3 compared to APOE4 mice.

Biological function	Protein description	Gene
**Fatty acid metabolism**	Acyl-coenzyme A synthetase, mitochondrial	*Acsm1*
	2-hydroxyacyl-CoA lyase 1	*Hacl1*
	Peroxisomal coenzyme A diphosphatase NUDT7	*Nudt7*
**Amino acid metabolism**	Asparagine synthetase [glutamine-hydrolyzing]	*Asns*
	Carbamoyl-phosphate synthase, mitochondrial	*Cps1*
	Cystathionine gamma-lyase	*Cth*
	3-hydroxyisobutyrate dehydrogenase, mitochondrial	*Hibadh*
	Ornithine aminotransferase, mitochondrial	*Oat*
	Serine--tRNA ligase, mitochondrial	*Sars2*
	Sodium-coupled neutral amino acid transporter 3	*Slc38a3*
**Cytoskeleton, intermediate filaments**	Keratin, type II cytoskeletal 1	*Krt1*
	Keratin, type II cytoskeletal 2 epidermal	*Krt2*
	Keratin, type II cytoskeletal 5	*Krt5*
	Keratin, type II cytoskeletal 7	*Krt7*
	Keratin, type I cytoskeletal 10	*Krt10*
	Keratin, type I cytoskeletal 16	*Krt16*
	Keratin, type I cytoskeletal 42	*Krt42*
	Keratin, type II cytoskeletal 72	*Krt72*
	Keratin, type II cytoskeletal 2 oral	*Krt76*
	Keratin, type II cytoskeletal 1b	*Krt77*
	Keratin, type II cytoskeletal 78	*Krt78*
	Keratin, type II cytoskeletal 79	*Krt79*
	Predicted pseudogene 5478	*Gm5478*
**Biotransformation, phase I and II**	Carboxylesterase 3B	*Ces3b*
	Cytochrome P450 2C44	*Cyp2c23*
	Cytochrome P450 2C29	*Cyp2c29*
	Cytochrome P450 2C37	*Cyp2c37*
	Cytochrome P450 2C 50	*Cyp2c50*
	Cytochrome P450 2C54	*Cyp2c54*
	NADPH-dependent 3-keto-steroid reductase Hsd3b5	*Hsd3b5*
	Glutathione transferase	*Mgst1*
	3-oxo-5-alpha-steroid 4-dehydrogenase	*Srd5a1*
	Sulfotransferase	*Sult2a8*
	UDP-glucuronosyltransferase 2A3	*Ugt2a3*
	UDP-glucuronosyltransferase 2B1	*Ugt2b1*
**Immune response**	Complement component C8 alpha chain	*C8a*
	D-dopachrome decarboxylase	*Ddt*
	Interferon-gamma-inducible GTPase Ifgga2 protein	*Gm4951*
	Ig gamma-2A chain C region, membrane-bound form	*Igh-1a*
	Regucalcin	*Rgn*
	Zymogen granule membrane protein 16	*Zg16*
**Pheromone binding**	Major urinary protein 1	*Mup1*
	Major urinary protein 25	*Mup3*
	Major urinary protein 5	*Mup8*
	Major urinary protein 1	*Mup22*
**Miscellaneous**	Carbonic anhydrase 5A, mitochondrial	*Ca5a*
	Claudin-3	*Cldn3*
	Ectonucleoside triphosphate diphosphohydrolase 5	*Entpd5*
	Heme-binding protein 1	*Hebp1*
	Glycine N-acyltransferase-like protein Keg1	*Keg1*
	N-acetyltransferase 8 (GCN5-related) family member 1	*Nat8f1*
	Ubiquitinyl hydrolase 1	*Otud6b*
	Peptidyl-prolyl cis-trans isomerase NIMA-interacting 4	*Pin4*
	Lithostathine-1	*Reg1*
	60S ribosomal protein L39	*Rpl39*
	Serine protease inhibitor A3K	*Serpina3k*
	Sodium/bile acid cotransporter	*Slc10a1*
	Solute carrier organic anion transporter family member 1A1	*Slco1a1*
	Thioredoxin domain-containing protein 15	*Txndc15*

### Many proteins with relevance to human NAFLD/NASH are significantly different in APOE3 mice

**Figure 5.**
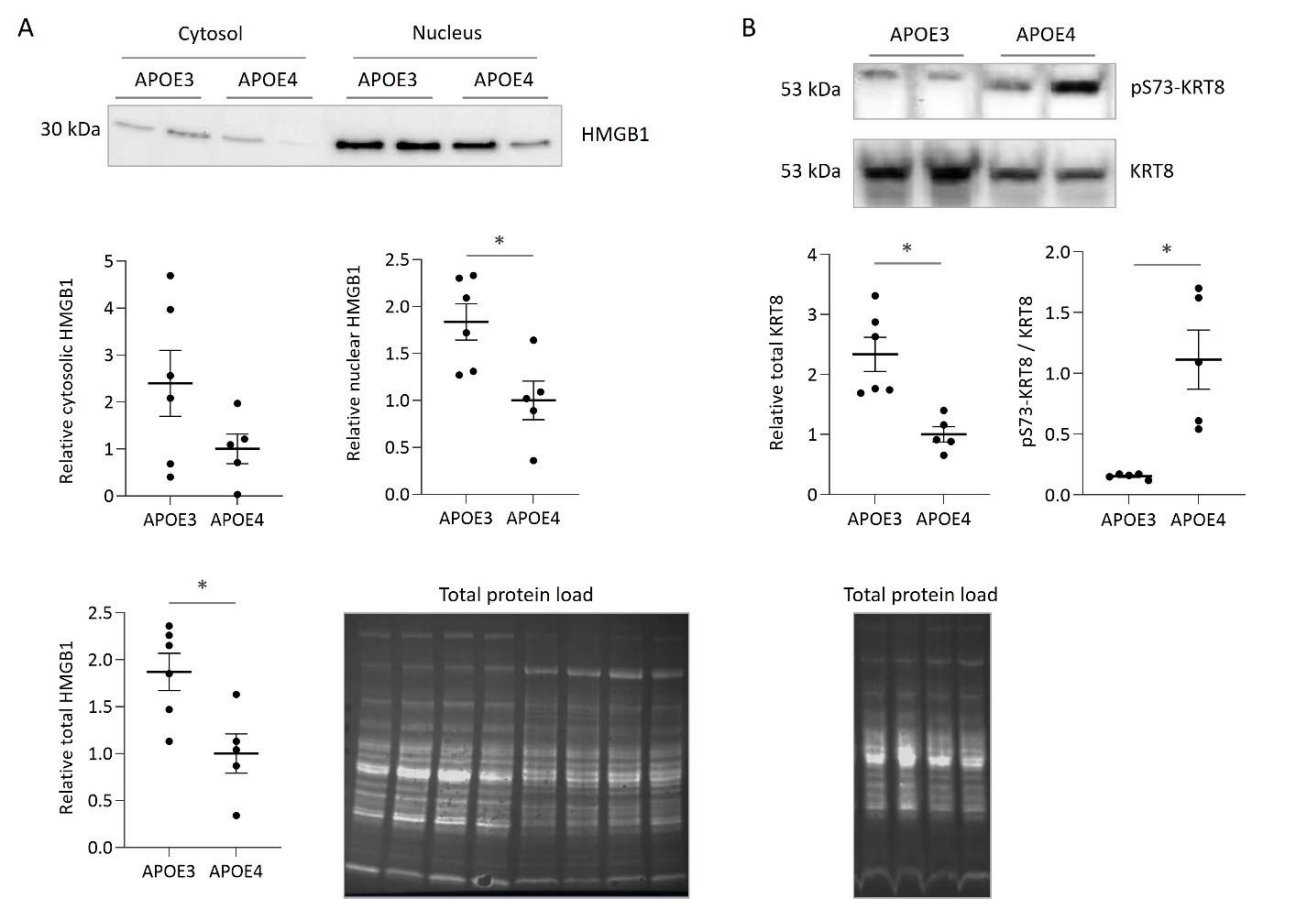
**Tissue damage response is induced and KRT8 turnover is altered in the liver of obese human APOE3 compared to APOE4 expressing mice**. Protein levels of HMGB1 (A) and KRT8 (B) were detected by Western blotting (WB) and representative images are shown. Target band intensity was related to total protein load visualized by the UV-induced reaction of trihalo compounds with tryptophan residues in proteins in the gel. Densitometric analysis was performed for all animals per group (APOE3, n=6; APOE4, n=5) and related to the mean band intensity of APOE4 mice for each WB. Individual data, the mean and the SEM are shown. Statistically significant (p<0.05) differences between APOE3 and APOE4 mice were identified by the Student’s t-test and indicated by an asterisk.

Numerous proteins differentially abundant in the liver of obese APOE3 mice have been already associated with either the risk, the disease progression or the clinical outcome of NAFLD in the literature (Table 5). We selected two targets from the list of differentially abundant proteins that have been suggested as markers of NASH-like disease pathology, namely the high mobility group B1 (HMGB1) protein and the keratin 8 (KRT8). We focused on cytosolic translocation of nuclear HMGB1 (Fig. 5A) and phosphorylation of KRT8 (Fig. 5B).

HMG proteins are enriched in the nucleus and involved in RNA processing, DNA repair or recombination. The translocation of nuclear HMGB1 to the cytosol and its subsequent secretion, is a marker of cell stress and tissue damage augmenting proinflammatory signaling [67, 68] and involved in NASH-like disease pathology [69]. Total hepatic and nuclear HMGB1 levels were significantly increased in our obese APOE3 mice as compared to APOE4. However, cytosolic translocation was not significantly different given the apparently high inter-individual variation in the APOE3 group (Fig. 5A).

The keratins 8 (KRT8) and 18 (KRT18) are typically co-expressed in epithelial cells such as hepatocytes and were higher abundant in obese APOE3 mice. Keratin turnover is regulated through ubiquitination and proteasomal degradation, which is attenuated by phosphorylation [70]. As determined by Western blotting, the levels of KRT8 phosphorylated at the serine residue 73 were significantly lower in APOE3 compared to APOE4 mice, while total KRT8 was increased (Fig. 5B). The calculated ratio of phosphorylated to total KRT8 was likewise significantly lower indicating a higher KRT8 turnover in obese APOE3 mice.

### Hepatic mRNA levels of genes involved in immune cell function and tissue damage response are elevated in APOE3 mice

**Figure 6.**
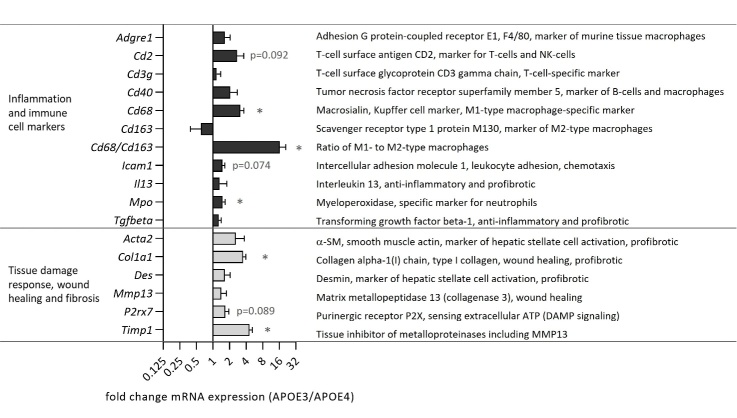
**Expression of immune cell markers is altered in the liver of obese human APOE3 compared to APOE4 expressing mice**. Relative mRNA levels of specific immune cell markers and tissue damage response genes were analyzed by qRT-PCR. Relative target genes were related to the expression of *Rn18S* (housekeeping gene) and calculated as fold change relative to the mean of APOE4 mice. The x-axis is given in log scale. Data are means + SEM (n=6 with the exception of a statistical outlier for *Cd2*, *Cd3g* and *MMP13*). An asterisk indicates a statistically significant (p<0.05) difference between APOE3 and APOE4 mice identified by the Student’s t-test or the Mann-Whitney U test (*Il13, Timp1*).

Relative mRNA levels of genes specific for immune cells or involved in tissue injury, wound healing and hepatic fibrosis have been determined in the liver of obese APOE3 and APOE4 mice. There is an overall induction of proinflammatory genes in obese APOE3 mice (Fig. 6), which is statistically significant for *Mpo* (encoding the neutrophil-specific marker myeloperoxidase) and *Cd68* (encoding the Kupffer cell- and M1-type macrophage-specific marker macrosialin). Furthermore, the ratio of M1- to M2-type macrophages, calculated as the individual ratio between relative *Cd68* and *Cd163* mRNA levels, was significantly elevated indicating a polarization shift to proinflammatory M1-type macrophages in the liver of obese APOE3 mice. The tissue damage response genes collagen type 1 (*Col1a1*) and the tissue inhibitor of metalloproteinases (*Timp1*) showed both significantly higher mRNA levels in APOE3 mice.

The induction of *Cd2* mRNA expression, a marker of T-cells and natural killer cells, did not reach statistical significance due to the very high interindividual variation (p=0.092). Likewise, the difference in mRNA levels of *Icam1*, a gene encoding a surface marker for leukocyte adhesion, and *P2rx7*, encoding the damage associate molecular pattern (DAMP) receptor P2X purinoceptor 7 for extracellular ATP, reached only borderline significance (p=0.074 and p=0.089, respectively).

## DISCUSSION

The human *APOE* gene is uniquely polymorph encoding three major protein variants including APOE3 (major variant), APOE4 and APOE2. Targeted replacement mice are engineered to express physiological levels of human APOE variants under the transcriptional control of endogenous APOE. Complementing associative data from humans [38-45], our study demonstrates that the human *APOEε3* genotype predisposes to fatty liver disease in targeted replacement mice, while *APOEε4* seems to be protective, when chronically subjected to overfeeding. Both the human APOE3 and APOE4 expressing mice developed severe adiposity, which was less pronounced in unmodified mice with similar genetic background (C57BL/6J). Along with DIO, the concentration of circulating biomarkers suggests an aggravated metabolic dysfunction in human APOE versus endogenous APOE expressing mice, which is in line with earlier studies [58, 59, 71]. There were no significant differences in those plasma biomarkers observable between APOE3 and APOE4 mice, which is also in line with previous reports [59, 72]. It is important to note though that VAT gene expression indicates a trend for increased adipose tissue inflammation in APOE3 versus APOE4 mice, which may have aggravated during prolonged HFSD feeding and increasing age of the mice.

Compared to others [58, 59], we observed 10-fold higher plasma APOE levels after long-term HFSD feeding, which, moreover, were significantly different between our APOE3 and APOE4 mice. The differences between our and earlier studies [58, 59] are the duration of the feeding being longer in the present study (7 vs. 2-3 months), and the duration of fasting before blood sampling (4 h vs. at least 16 h). It is known that human APOE secretion rises upon high-fat diet feeding [58] and that APOE expression in adipocytes promotes lipid accumulation and adiposity [73-75]. Furthermore, human APOE3 in mice preferably associates with triglyceride-rich VLDL particles [58], which is the main fraction of postprandial lipoproteins hours after meal ingestion [76]. Therefore, we hypothesize that the comparatively higher circulating APOE levels in our mice were due to the shorter fasting period (still circulating APOE-containing VLDL particles to be cleared) and longer duration of HFSD feeding and DIO development (generally elevated plasma lipids including APOE). However, the twofold elevated plasma concentration in APOE3 vs. APOE4 mice is surprising, since hepatic protein expression and mRNA levels in the liver and adipose tissue were similar between the mice. Since total plasma triglycerides and cholesterol levels were similar between APOE3 and APOE4 mice, the concentration of APOE per lipoprotein particle may have been altered in APOE3 mice. However, this remains to be elucidated in further studies.

**Table 5:** Examples of proteins with relevance to NAFLD/NASH in humans and in animal models that are significantly higher (↑) or lower (↓) abundant in our APOE3 compared to APOE4 mice.

Gene	Description	Abundance in APOE3 mice	Relevance/reference to NAFLD/NASH
***AldoB***	Fructose-bisphosphate aldolase	↑	Increased in plasma of NAFLD patients and in a murine NAFLD model [104]
***Anxa2/5***	Annexins A2/A5	↑	Significant increase of ANXA2 in hepatic expression with NAFLD and NASH, linked to 14-3-3 proteins and associated with poor clinical outcome [105] Hepatic ANXA5 expression correlates with disease progression from NAFLD to NASH [106]
***Col14a1***	Collagen alpha-1(XIV) chain	↑	High hepatic *COL4A1* expression is associated with disease progression from NAFLD to NASH [106]
***Cyp2c23/ 29/37/ 44/50/54***	Cytochrome P450 family 2c, polypeptides 23, 29, 37, 44, 50, 54	↓	Negative association of hepatic *CYP2C19* expression with disease progression from NAFLD to NASH [106]
***C8a***	Complement component C8 alpha chain	↓	Negative association of the C8 gamma chain with NASH progression and advanced fibrosis in human plasma [107]
***Fabp4***	Fatty acid binding protein-4	↑	High hepatic FABP4 expression is associated with ectopic lipid deposition [108], and considered a predictive biomarker of NAFLD/NASH disease progression [106]
***Fcer1g***	High affinity immunoglobulin epsilon receptor gamma	↑	Increased polymeric immunoglobulin receptor PIGR in plasma of NAFLD patients and a murine NAFLD model [104]
***Hmga1*, *Hmgn2 Hmgb1***	High mobility group (HMG) proteins AT-hook protein 1 (A1), nucleosome-binding domain-containing protein 2 (N2), B1	↑	HMG proteins are associated with inflammation and DAMP response [109] Increased level of hepatic [69, 110] and circulating HMGB1 [111] in response to diet-induced hepatocyte injury and fibrosis and as part of NASH-like disease pathology, respectively
***Lgals3***	Galectin-3	↑	Increased *Lgals3* liver expression in steatohepatitis [112], LGALS3 plays a key role in tissue fibrosis [113] Positive association with plasma levels of galectin-3 binding protein (LGALS3BP) in NAFLD patients [104] and a subgroup with advanced fibrosis [114]
***Oat***	Ornithine aminotransferase	↓	Negative association with disease progression from NAFLD to NASH [106]
***Pin4***	Peptidyl-prolyl cis-trans isomerase NIMA-interacting 4 (parvulin-14)	↓	Downregulated in rat liver with NASH phenotype [115], key regulator of the prevention of hyperlipidemia and obesity in mice [116]
***Plin3/4***	Perilipin-3 and 4	↑	Hepatic *PLIN1* and *PLIN2* expression is increased in NAFLD and NASH [105] *Plin3* and *Plin4* are association with hepatic lipid accumulation in mice [117], *Plin4* is downregulated through dietary intervention preventing hepatic steatosis [117]
***Rarres2***	Chemerin	↑	Hepatic and circulating levels are higher in NAFLD patients, positive associated with NASH activity score, hepatocyte injury, degree of steatosis and hepatocellular carcinoma [118]
***Sult2a8***	Sulfotransferase family 2A, dehydroepiandrosterone (DHEA)-preferring, member 8	↓	SULT protein level (1A1, 2A1) and activity reduced in the liver of NASH patients [119]
***S100a1/10/11***	S100 calcium binding proteins A1/A10/A11	↑	S100a proteins are involved in the transition from steatosis to fibrosis and hepatocellular carcinoma [120-122], positive association of S100A11 with disease progression in a tree shrew NAFLD model promoting hepatic lipid accumulation [120], *S100a10* is upregulated in hepatic steatosis and downregulated through dietary intervention normalizing the metabolic dysregulation in mice [117],
***Tubb6***	Tubulin beta-6 chain	↑	Significant increase in hepatic expression of *TUBA1A* in NAFLD and NASH, linked to 14-3-3 proteins and associated with poor clinical outcome [105], upregulated in mice with hepatic steatosis and downregulated through dietary intervention normalizing the metabolic dysregulation [117]
***Vim***	Vimentin	↑	upregulated in the liver of mice with NASH phenotype [123-125] and associated with the severity of liver steatosis [90]
***Ywhah***	14-3-3 protein eta	↑	*YWHAZ* and *YWHAH* orchestrate metabolic and inflammatory dysregulated genes distinguishing patients from healthy controls, 14-3-3 proteins play an important role in disease progression from NASH to NAFLD to hepatocellular cancer [105]

Postprandial triglycerides can be cleared from the plasma for storage in metabolically active subcutaneous adipose tissue (SAT). Opposed to this nonectopic storage, ectopic lipid deposition is defined as adipose tissue surrounding organs, such as the visceral adipose tissue, or within organs, such as hepatic steatosis, which is considered metabolically adverse [77-79]. Though nonsignificant, we have observed an increase of relative SAT volumes in APOE4 mice indicating a higher capacity of beneficial nonectopic fat deposition. This fits well with data from others showing that the *APOEε4* genotype is associated with less VAT compared to their *APOEε3* homozygous counterparts in obese human subjects and targeted replacement mice [72, 80-82]. Although our mice exhibited similar total VAT volumes, there was a massive ectopic fat accumulation in the liver of APOE3 mice, which would be in line with the saturation of metabolically beneficial nonectopic lipid deposition in the SAT compared to APOE4 mice. Consistently, we observed an inverse relationship between ectopic fat accumulation in the liver and the relative amount of SAT in our human APOE expressing mice. In contrast to the percentage of SAT, final body weight was clearly not a determinant of hepatic steatosis in the present study.

Although APOE isoform-dependent effects on hepatic steatosis have not been investigated in mouse models before, APOE deficient mice not only develop atherosclerosis but also NASH pathology including liver steatosis and fibrosis upon high-fat diet feeding [83-85]. Interestingly, in genetically obese mice additional APOE deficiency prevents severe adiposity and ectopic lipid deposition in the liver [86, 87] emphasizing the complexity of the interaction of APOE with obesity, ectopic and nonectopic lipid deposition and related pathologies. Despite the lack of significance in the present study, it cannot be ruled out that the concentration of plasma APOE affects the development of fatty liver disease. The question of whether fasted and postprandial circulating APOE is a determinant of NAFLD and NASH may be investigated in humans under consideration of their *APOE* genotype status in the future.

Analyzing the liver proteome, we found an unexpectedly high number (164) of differentially abundant proteins between APOE3 and APOE4 mice. To the best of our knowledge, there is no comparable study focusing on the *APOE* genotype-dependent effect on the whole cell hepatic proteome. However, the effect of high-fat diet feeding and fatty liver induction on hepatic protein abundance in laboratory rodents has been investigated before identifying between 12 and 96 significant proteins [88-93]. Several of those highly fatty liver-associated proteins [90] were also higher abundant in our APOE3 mice including cytokeratin 8 and 18, vimentin or annexin A5. On the other hand, proteins that were higher abundant in our APOE4 compared to APOE3 mice have been associated with healthy livers on control diets such as glutathione-S-transferase and major urinary proteins [93]. Therefore, we suggest that the majority of differentially abundant proteins are more related to the development of a NASH-like phenotype in APOE3 mice than to the *APOE* genotype per se. Given that animal models for human NASH are allegedly insufficient in displaying the whole spectrum of pathological conditions [94], it is intriguing that we observed quite a few clinical signs and prerequisites of human NAFLD and NASH in our APOE3 expressing mice (please see also Table 5). For example, hepatic *Mpo* mRNA expression was increased, which is indicative of neutrophil invasion and has been considered a key event in the progression of hepatic steatosis to NASH [95, 96]. The increasing presence of MPO positive cells leads to the activation of hepatic stellate cells and drives fibrosis through collagen production [97]. In accordance, we observed evidence for hepatocyte injury, inflammation and initial fibrosis in APOE3 expressing mice including signs of increased KRT8 turnover and increased expression of damage response genes.

The underlying mechanisms leading to the higher abundance of steatosis-inducing proteins, however, remain obscured. Recently, the role of the lectin galectin-1 (*Lgals1*) in hepatic lipid deposition and inflammation has been investigated [98]. *Lgals* knockout mice exhibited significantly lower ectopic lipid deposition and downregulated lipogenic and inflammatory genes expression compared to wildtype mice, but galectin-1 expression was increased upon high-fat diet feeding promoting obesity through enhanced peroxisome proliferator activated receptor gamma activation [98]. These findings may be of great relevance in helping to explain the propensity of APOE3 mice to develop fatty liver disease. Galectins-1 and 3 as well as lipogenic proteins such as fatty acid binding proteins 4 and 7, perilipins 3 and 4, and chemerin were all higher abundant in the liver of our APOE3 mice. Since galectins are part of the innate immunity supporting pathogen recognition it is also highly important to consider that endotoxemia induces hepatic inflammation and injury, which is relevant in human NASH [94]. In addition to galectins, we observed a higher level of endotoxin sensing in APOE3 compared to APOE4 mice including higher protein levels of the circulating lipopolysaccharide (LPS) binding protein LBP and the pathogen recognition receptor CD14. Furthermore, hepatic mRNA levels of the LPS-activated toll-like receptor 4 (*Tlr4*) and its downstream targets tumor necrosis factor alpha (*Tnfa*) and interleukin 6 (*Il6*) were increased in our APOE3 mice. One possible explanation for the higher systemic endotoxin load in APOE3 mice may be the lower intestinal LPS detoxification capacity evident as compared to APOE4 mice. These data can be viewed in a previous independently published study that focused on the interaction of the *APOE* genotype with innate immune sensing [32]. Taken together, it may be suggested that dysbiosis and endotoxemia related to HFSD feeding is more advanced in the presence of APOE3 than APOE4, which promoted hepatic pathogen recognition and lipogenesis and contributed to fatty liver disease progression in our mouse model.

Our data picturing APOE4 as a protecting factor against hepatic steatosis and steatohepatitis under long-term high-fat overfeeding conditions promote the paradigm shift on the APOE-disease risk associations [28]. For more than three decades, *APOE epsilon* 4 has been recognized as solely adverse in terms of morbidity and mortality in the elderly [14, 15, 18, 99, 100], until the discovery of potentially superior health effects compared to *APOE epsilon 3* [23, 101, 102], for example a higher vitamin D level in geographical regions of insufficient endogenous synthesis [29]. The negative NAFLD risk association suggested in the present study is in sharp contrast to the increased AD and CVD risk also related to the *APOE epsilon 4* allele. Since the etiology of those disorders is complex involving multiple different biochemical and histological hallmarks, a comprehensive explanation of this supposed contradiction seems almost impossible. At least, it may be of significance to note that APOE has been emphasized to exhibit differential cellular effects in the brain as opposed to the liver [103]. For example, while brain ATP levels are actually lower in APOE4 than APOE3 expressing mice [50], no differences have been observed in the liver [56]. It is also conceivable that similar to the pleiotropic nature of the *APOE* gene during lifetime, meaning that one given trait is beneficial during early years while being adverse later in life, a certain molecular trait of the APOE4 protein isoform maybe protective or neutral in the liver but harmful in the brain. Our present data indicate that the higher capacity of nonectopic lipid storage along with lower hepatic pathogen recognition and subsequent lower upregulation of lipogenic and inflammatory pathways compared to APOE3 may be involved in the NASH-preventing activity of APOE4 in targeted replacement mice. Understanding the underlying mechanisms of *APOE* genotype-dependent disease pathology will contribute to the development of future therapeutic treatment options.
